# Supplementary material for: Novel Dairy Fermentates Have Differential Effects on Key Immune Responses Associated with Viral Immunity and Inflammation in Dendritic Cells
Source: Foods. 2024 Jul 29;13(15):2392. doi: 10.3390/foods13152392 (PMC11311654; doi:10.3390/foods13152392)
Supplement: Supplementary file 1 [file foods-13-02392-s001.zip › foods-3120600-supplementary.pdf]

Table S1: Mean and Confidence intervals for cytokines in the presence of LOX, or LPS activated BMDCs, stimulated with fermentates.

	IL-10		TNF		IL-1	
		0±0		0±0		2.504±0
		83±13.415		816.3±189.8		2587±341
		46.7±14.505		1188±210		197.6±83.15
		0±0		0±0		68.47±23.345
		212.2±14.95		2349±226.5		2766±693.5
		111.9±47.475		2989±172.5		310.2±14.7
		149.9±67.85		792.3±169.1		63.05±1.635
		295.5±33.4		2199±786.5		1428±130
		326.7±28.3		4286±211.5		163.5±42.8
		0±0		160±90.3		58.35±44.145
		776.7±65.3		736.6±15.1		3574±508
		188.9±99.6		826±127.5		325.3±52.45
		2218±240.5		892.8±111.5		802.7±200
		2685±149		931.5±213.35		1537±196
		2549±130.5		828.5±88.8		818.4±225.5
		1648±271		1123±133.3		803.6±55.85
		1965±108		938.8±172.55		1181±87.5
		2152±231.5		1016±88.7		1218±270.5
		0±0		0±0		3.417±0.6925
		95.83±43.485		2635±693		1678±140
		0±0		3005±329.5		72.49±13.485
		0±0		487.5±177.35		85.62±12.39
		255.5±45.85		2962±341		1077±87
		180.4±23.3		3063±322		301.5±82.75
		38.46±4.445		1256±173		40.48±12.095
SC229 + LOX	362.3	362.3±169.35	2749	2749±320.5	662	662±47.65
SC229 + LPS	272.8	272.8±16.4	3856	3856±644	149.6	149.6±18.15

IL-6		IL-12p40		IL-12p70		IL-23		IL-27	
**Mean**	**Confidence interval (95%CI)**	**Mean**	**Confidence interval ((5%CI)**	**Mean**	**Confidence interval (95%CI)**	**Mean**	**Confidence interval (95%CI)**	**Mean**	**Confidence interval (95%CI)**
182.7	182.7±4.05	244.9	244.9±51.8	0	0±0	0	0±0	9.392	9.392±3.3285
4949	4949±306.5	1586	1586±333	0	0±0	39.46	39.46±32.8	108.4	108.4±52.475
8462	8462±565.5	1989	1989±682.5	0	0±0	147.5	147.5±85.735	1181	1181±189.55
925.1	925.1±275.95	219.9	219.9±8.2	0	0±0	0	0±0	99.78	99.78±19.41
11583	11583±1050.5	3652	3652±299	170	170±29.5	379.2	379.2±127.8	571.7	571.7±75.8
8859	8859±740	903.6	903.6±75.1	0	0±0	265	265±47.7	1167	1167±29.5
3092	3092±320	402.2	402.2±93.75	0	0±0	0	0±0	153.9	153.9±62.6
7583	7583±1449	2201	2201±376.5	0	0±0	89.74	89.74±36.275	177.6	177.6±26.75
11741	11741±1533	899.2	899.2±190.15	23.43	23.43±3.315	138.7	138.7±21.3	949.8	949.8±56.2
3264	3264±2461.685	7358	7358±4495.5	0	0±0	47.69	47.69±33.09	110.2	110.2±128.32
11998	11998±3002.5	26700	26700±1860	112.2	112.2±39.665	415.9	415.9±166.75	345.6	345.6±71.6
13703	13703±1543.5	16925	16925±2030	16.94	16.94±6.26	72.84	72.84±6.985	1306	1306±134
14477	14477±1868.5	8722	8722±1605.5	2.052	2.052±30775	276.5	276.5±12.65	1140	1140±100.5
22066	22066±1821.5	15845	15845±630.5	120.3	120.3±33.055	358.8	358.8±11.3	557.3	557.3±154.6
17608	17608±869	8932	8932±1068	15.73	15.73±23.6	442.2	442.2±12.55	1415	1415±291.5
15626	15626±3166.5	14859	14859±4195.5	20.55	20.55±13.2775	368.8	368.8±50.05	1297	1297±107
19669	19669±3412	18085	18085±2637	97.97	97.97±16.865	473.6	473.6±69.7	439.5	439.5±73.55
16835	16835±1898	12703	12703±1065	45.13	45.13±29.305	603.7	603.7±70.5	1306	1306±236.5
0	0±0	0	0±0	3.389	0±0	0	0±0	0	0±0
4198	4198±1019.5	2666	2666±583.5	13.23	13.23±8.3905	72.32	72.32±12.785	54.06	54.06±53.935
5621	5621±781	1200	1200±345.05	14.52	14.52±3.66	63.52	63.52±16.435	1323	1323±77
775.1	775.1±285.65	178.7	178.7±101.89	3.519	3.519±1.846	0	0±0	0	0±0
7182	7182±218	5145	5145±261	83.69	83.69±9.75	150.8	150.8±23.1	474.6	474.6±77.75
6372	6372±1069.5	1155	1155±242.7	70.83	70.83±27.06	187.8	187.8±101.3	1167	1167±225.25
1214	1214±169	883.8	883.8±198.25	45.36	45.36±26.815	24.45	24.45±9.23	70.31	70.31±3.215
7528	7528±1566	4956	4956±442.5	141.5	141.5±60.58	240.1	240.1±40.15	411.9	411.9±116.7
7336	7336±1034	2380	2380±208	176.1	176.1±26.9	271	271±62.9	1320	1320±94.5

Table S2: Raw metabolomics data.

Sample ID	Strain	Acetate	Lactate	2-Aminobu	2-Hydroxyi
SC232-A	SC232	0.3799	4.5561	0.0004	0.0121
SC232-B	SC232	0.3938	4.6896	0.0006	0.014
SC232-C	SC232	0.4857	5.0648	0.0003	0.0137
SC232-D	SC232	0.4436	5.0608	0.0001	0.0138
SC232-E	SC232	0.3982	4.7441	0.0004	0.0112
SC232-F	SC232	0.4279	4.8468	0.0008	0.0132
SC40-A	SC40	0.0759	7.0487	0.0053	0.0182
SC40-B	SC40	0.0766	6.9159	0.0057	0.0212
SC40-C	SC40	0.0759	7.0287	0.0051	0.0189
SC40-D	SC40	0.0836	7.5778	0.0044	0.0208
SC40-E	SC40	0.0698	6.553	0.0078	0.02
SC40-F	SC40	0.0717	6.5692	0.0037	0.0167
SC218-A	SC218	0.7914	3.6625	0.0007	0.012
SC218-B	SC218	0.8202	3.3157	0.0004	0.0101
SC218-C	SC218	0.752	3.4176	0.0005	0.012
SC218-D	SC218	0.844	2.8331	0.0003	0.0115
SC218-E	SC218	0.7663	3.5903	0.0005	0.0103
SC218-F	SC218	0.8395	3.4901	0.0007	0.0118
SC212-A	SC212	0.5048	9.7422	0.0013	0.027
SC212-B	SC212	0.8337	14.7752	0.0043	0.0333
SC212-C	SC212	0.606	10.5137	0.0018	0.0221
SC212-D	SC212	0.5694	10.3537	0.0038	0.0338
SC212-E	SC212	0.7724	13.8341	0.0053	0.0249
SC212-F	SC212	0.5637	10.5519	0.0034	0.0221
SC215-A	SC215	0.0396	5.0445	0.0005	0.0008
SC215-B	SC215	0.0344	4.4289	0.0003	0.0004
SC215-C	SC215	0.0357	4.7551	0.0004	0.0011
SC215-D	SC215	0.0387	4.9131	0.0003	0.0012
SC215-E	SC215	0.0423	5.014	0.0002	0.0012
SC215-F	SC215	0.0376	4.8513	0.0003	0.0011
RSMVE-A	RSMVE	0.0161	0.0065	0.0008	0.0032
RSMVE-B	RSMVE	0.0182	0.0125	0.001	0.0026
RSMVE-C	RSMVE	0.0204	0.0075	0.0008	0.0032
RSMVE-D	RSMVE	0.0107	0.0026	0.0002	0.0004
RSMVE-E	RSMVE	0.016	0.0053	0.0007	0.0027
RSMVE-F	RSMVE	0.0133	0.0085	0.0002	0.0005

2-Hydroxyisovalerate	2-Oxoglutarate	4-Aminobutyrate	5-Phenylvaleric acid
0.0041	0.0316	0.0042	0.0036
0.0054	0.0266	0.002	0.0079
0.0057	0.0343	0.0033	0.0064
0.0045	0.0086	0.0003	0.002
0.0047	0.0319	0.0034	0.0049
0.0043	0.0348	0.0062	0.0027
0.0048	0.0042	0.0071	0.0148
0.0049	0.0035	0.0061	0.02
0.0048	0.003	0.0042	0.016
0.0052	0.0051	0.008	0.0113
0.0045	0.0052	0.008	0.0203
0.0043	0.0043	0.0052	0.0143
0.0027	0.0147	0.0046	0.002
0.0025	0.0133	0.0047	0.0037
0.0026	0.013	0.0044	0.0027
0.0022	0.0113	0.0023	0.0052
0.0026	0.0113	0.0044	0.0058
0.0029	0.0147	0.0053	0.0064
0.02	0.085	0.0062	0.0102
0.0305	0.137	0.0096	0.0089
0.0204	0.089	0.0041	0.0029
0.0194	0.0864	0.0052	0.0056
0.0272	0.1282	0.0071	0.014
0.0201	0.0874	0.005	0.0075
0.001	0.0069	0.006	0.0035
0.0007	0.0093	0.0071	0.0023
0.0007	0.0088	0.0066	0.0044
0.0006	0.0089	0.0072	0.0039
0.0006	0.0094	0.0074	0.0037
0.0006	0.0106	0.0058	0.0029
0.001	0.0058	0.0038	0.003
0.0015	0.0041	0.0033	0.004
0.0013	0.0044	0.0024	0.0036
0.0002	0.0029	0.0025	0.0005
0.0007	0.0052	0.0041	0.0022
0.0001	0.003	0.0021	0.0008

Acetoin	Acetone	Alanine
0.0021	0.0027	0.0293
0.0017	0.002	0.0313
0.0022	0.0031	0.0325
0.0004	0.0025	0.0327
0.0018	0.0016	0.0309
0.0026	0.0027	0.0315
0.0024	0.0025	0.0056
0.0022	0.0021	0.0059
0.002	0.0021	0.006
0.0027	0.0053	0.0059
0.0028	0.0027	0.0056
0.0021	0.0031	0.0047
0.1094	0.0042	0.0038
0.1065	0.0018	0.0037
0.1023	0.0022	0.0036
0.1001	0.0018	0.0033
0.1047	0.0014	0.0036
0.1084	0.0016	0.0044
0.0023	0.0019	0.0955
0.0037	0.0036	0.1433
0.0014	0.002	0.1018
0.0019	0.0022	0.1001
0.0026	0.0032	0.1272
0.0019	0.0023	0.0982
0.0262	0.0013	0.0047
0.0217	0.0022	0.0039
0.0268	0.0014	0.0045
0.0304	0.0015	0.0042
0.029	0.003	0.0038
0.0216	0.0016	0.0048
0.0013	0.0027	0.0029
0.0013	0.0026	0.0032
0.0011	0.0028	0.0032
0.0032	0.0015	0.0013
0.0012	0.0025	0.0027
0.0004	0.0016	0.0018

Benzoate	Betaine	Butyrate	Choline
0.0004	0.011	0.0069	0.039
0.0005	0.0107	0.0072	0.0413
0.0007	0.0112	0.0083	0.0424
0.0011	0.0087	0.0045	0.036
0.0005	0.0119	0.0069	0.0433
0.0004	0.0121	0.0081	0.0419
0.0054	0.0079	0.0071	0.0331
0.0061	0.0087	0.0068	0.0359
0.0068	0.0083	0.0063	0.0359
0.0056	0.009	0.0065	0.0372
0.0055	0.0092	0.0061	0.0384
0.0051	0.0072	0.0068	0.0304
0.0048	0.0101	0.0073	0.0437
0.0051	0.0091	0.0069	0.0373
0.0053	0.0089	0.0071	0.0398
0.006	0.0089	0.006	0.0362
0.0072	0.0085	0.0063	0.0374
0.0065	0.0104	0.0082	0.0462
0.0069	0.0086	0.0079	0.0395
0.0054	0.0112	0.0113	0.0551
0.0063	0.0089	0.004	0.0404
0.0066	0.0074	0.0062	0.0385
0.0071	0.0106	0.0073	0.0486
0.0062	0.0075	0.0075	0.0364
0.0017	0.0094	0.0071	0.0408
0.0016	0.009	0.0068	0.0364
0.0019	0.0107	0.0068	0.0431
0.002	0.0096	0.0073	0.039
0.0016	0.0084	0.0077	0.0375
0.0015	0.0091	0.0071	0.0376
0.0004	0.0099	0.0094	0.0416
0.0005	0.0104	0.0091	0.0468
0.0005	0.0108	0.0098	0.0391
0.0003	0.0071	0.0053	0.0273
0.0003	0.011	0.0096	0.0453
0.0004	0.0084	0.0062	0.0345

Citrate	Creatine	D-Lactic acid	Desaminotyrosine
0.2723	0.0199	0.0183	0.0002
0.3178	0.0203	0.019	0.0002
0.2418	0.0238	0.0207	0.0002
0.3061	0.0182	0.0208	0.0001
0.272	0.0214	0.0191	0.0002
0.2728	0.0209	0.0199	0
0.4398	0.015	0.028	0.0045
0.4362	0.0154	0.0276	0.0032
0.4518	0.0157	0.0277	0.0018
0.4698	0.0179	0.0299	0.0059
0.4601	0.0163	0.0266	0.0051
0.409	0.014	0.0261	0.0035
0.0176	0.0241	0.0148	0.0012
0.0083	0.0218	0.0141	0.0013
0.0048	0.0224	0.0143	0.0013
0.0043	0.021	0.0122	0.0018
0.0052	0.022	0.0146	0.0018
0.0101	0.0232	0.0154	0.0015
0.0907	0.0163	0.0395	0.009
0.098	0.0246	0.0595	0.011
0.0665	0.0183	0.0426	0.0062
0.068	0.017	0.0433	0.0044
0.0985	0.0229	0.0522	0.0049
0.089	0.017	0.0413	0.0031
0.49	0.0204	0.0202	0.0005
0.4201	0.0186	0.0174	0.0006
0.5038	0.0197	0.0191	0.0006
0.4766	0.0205	0.0198	0.0008
0.4701	0.0197	0.0196	0.0006
0.4772	0.0205	0.019	0.0006
0.5035	0.0256	0.0016	0.0002
0.5725	0.0302	0.0016	0.0005
0.5674	0.0325	0.0016	0.0002
0.3162	0.0178	0.0007	0.0001
0.5235	0.0267	0.0016	0.0003
0.3251	0.0215	0.0007	0

Dihydroxyacetone	Ethanol	Formate	Fumarate
0.2245	0.0739	0.0422	0.0008
0.231	0.0762	0.0423	0.0007
0.2645	0.0903	0.024	0.0009
0.2285	0.0788	0.0498	0.0012
0.2385	0.0797	0.0261	0.0008
0.2425	0.0822	0.0453	0.0007
0.0876	0.0619	0.0163	0.0001
0.0823	0.0654	0.0179	0.0001
0.083	0.0644	0.0189	0.0001
0.0968	0.0685	0.0178	0
0.0825	0.0609	0.0138	0
0.0822	0.0588	0.0142	0
0.0369	0.1353	0.1121	0.0001
0.0364	0.1283	0.1074	0.0002
0.0315	0.126	0.1123	0.0001
0.0258	0.126	0.0428	0.0003
0.0276	0.1311	0.1116	0.0004
0.0338	0.1318	0.0881	0.0001
0.1917	0.0872	0.0235	0.0003
0.3114	0.1406	0.0388	0.0001
0.2191	0.1002	0.0287	0.0001
0.1934	0.0873	0.0122	0.0001
0.2904	0.1311	0.0336	0.0004
0.2053	0.0942	0.0241	0.0002
0.0314	0.1492	0.0665	0.0101
0.0312	0.1287	0.0543	0.0085
0.0321	0.1462	0.0599	0.0096
0.0329	0.1544	0.0664	0.0105
0.0289	0.1325	0.0586	0.01
0.0336	0.1477	0.0639	0.0102
0.0421	0.0132	0.0619	0.0014
0.0396	0.0174	0.0812	0.0018
0.0414	0.0178	0.0868	0.0018
0.0255	0.0092	0.0457	0.001
0.0407	0.0143	0.0667	0.0015
0.0276	0.0109	0.0378	0.0013

Galactose	Glucose	Glutamate	Glycerophosphocholine
0.1175	0.0362	0.0139	0.3731
0.1371	0.0134	0.012	0.3889
0.1318	0.0305	0.0129	0.4264
0.0302	0.017	0.0034	0.3564
0.1301	0.0235	0.0125	0.4192
0.1171	0.0429	0.0196	0.3947
2.7659	0.1389	0.0733	0.3198
2.167	0.1371	0.0784	0.3386
3.0632	0.1248	0.0786	0.3376
2.7158	0.1684	0.0803	0.363
3.0791	0.1636	0.0842	0.361
2.8472	0.1399	0.0703	0.2873
0.0061	0.0289	0.0427	0.4174
0.0048	0.0454	0.05	0.3701
0.0051	0.0183	0.0366	0.3831
0.0044	0.016	0.0348	0.3418
0.0067	0.0212	0.0359	0.36
0.0011	0.0198	0.0427	0.4267
0.1506	1.3191	0.0556	0.3687
0.2093	1.6208	0.0598	0.5097
0.1441	1.293	0.0327	0.373
0.1453	1.1602	0.0503	0.3574
0.1955	1.3635	0.06	0.4744
0.1429	1.2288	0.0356	0.3405
2.1534	0.022	0.0064	0.3952
1.3726	0.0265	0.0095	0.3457
2.2191	0.0236	0.0074	0.4014
2.2634	0.0436	0.0076	0.3882
1.3729	0.0459	0.0072	0.3757
1.9803	0.0424	0.0082	0.3676
0.1187	0.0386	0.0196	0.4387
0.1442	0.0354	0.0181	0.4884
0.1388	0.036	0.0189	0.4949
0.0969	0.0277	0.0112	0.2722
0.1307	0.0453	0.0197	0.4627
0.0985	0.0293	0.0124	0.3523

Hydroxyacetone	Isoleucine	Lactose
0.0032	0.0006	4.5253
0.003	0.0005	4.9412
0.0032	0.0005	5.0888
0.002	0.0003	4.7594
0.003	0.0001	5.1445
0.0038	0.0007	4.8841
0.0042	0.0056	2.337
0.0043	0.0061	2.7101
0.0039	0.0059	2.6208
0.0045	0.0061	2.5202
0.0051	0.0061	2.789
0.0041	0.0054	2.4237
0.0049	0.001	5.2253
0.0027	0.0008	4.841
0.0025	0.0006	4.8704
0.0021	0.0006	4.5204
0.0026	0.001	4.2542
0.003	0.0009	5.3964
0.0019	0.0079	3.0439
0.0035	0.0148	4.1875
0.0014	0.0082	2.9459
0.002	0.0073	2.8293
0.0022	0.0121	3.7201
0.002	0.0078	2.8702
0.0017	0.0012	4.0804
0.002	0.0014	3.4526
0.0018	0.0013	4.3084
0.0018	0.0011	3.8732
0.0019	0.0009	3.6638
0.0019	0.0012	3.5713
0.0024	0.0005	6.3672
0.0023	0.0005	6.9861
0.0023	0.0005	7.2684
0.0015	0.0003	3.8302
0.0025	0.0005	6.6298
0.0016	0.0002	5.4621

Lactulose	Leucine	Lysine	Malonate	
	0.2052	0.0017	0.0052	0.0122
	0.2363	0.003	0.0025	0.01
	0.2089	0.0012	0.0035	0.0125
	0.2755	0.0009	0.003	0.0022
	0.2386	0.0016	0.0033	0.0121
	0.2224	0.0025	0.0101	0.0141
	0.1569	0.0118	0.0031	0.0111
	0.1717	0.0157	0.0031	0.0102
	0.1653	0.016	0.0031	0.0089
	0.1678	0.0137	0.0049	0.013
	0.172	0.0144	0.0085	0.0131
	0.1514	0.0157	0.0054	0.01
	0.1953	0.0039	0.0091	0.0141
	0.1675	0.0035	0.0076	0.0128
	0.167	0.0037	0.0057	0.0121
	0.1688	0.0028	0.0083	0.0106
	0.148	0.0032	0.0063	0.0118
	0.223	0.0045	0.0101	0.0133
	0.2156	0.0161	0.0107	0.0122
	0.2527	0.0304	0.0174	0.0185
	0.1826	0.0126	0.0118	0.014
	0.1915	0.0163	0.0073	0.0108
	0.2726	0.0228	0.0065	0.0155
	0.1785	0.0158	0.0047	0.0111
	0.2297	0.0014	0.0014	0.01
	0.1833	0.0005	0.0028	0.0114
	0.2091	0.0005	0.0036	0.0113
	0.2064	0.0008	0.0015	0.0111
	0.1467	0.0005	0.0041	0.0111
	0.1711	0.0006	0.0024	0.0118
	0.2446	0.0016	0.0032	0.0134
	0.283	0.0022	0.0018	0.0135
	0.2879	0.0014	0.002	0.0139
	0.1538	0.0003	0.0031	0.0089
	0.2723	0.0015	0.0036	0.0139
	0.2271	0.0003	0.0024	0.0101

Methanol	Orotic acid	Phenylacetate	Phenylalanine
0.0023	0.1417	0.0024	0.0002
0.001	0.1402	0.0023	0.0002
0.0012	0.1582	0.0023	0.0004
0.0035	0.1669	0.0015	0.0005
0.0019	0.1561	0.0028	0.0002
0.0025	0.1457	0.0027	0.0002
0.0047	0.0058	0.006	0.0016
0.0039	0.0045	0.0029	0.0049
0.0036	0.0037	0.0053	0.0058
0.0058	0.0053	0.0053	0.0044
0.0054	0.0054	0.0037	0.0046
0.0041	0.004	0.0035	0.0044
0.0022	0.1176	0.0005	0.0003
0.0025	0.1196	0.0004	0.0004
0.0019	0.1148	0.0008	0.0004
0.0018	0.1163	0.001	0.001
0.0014	0.1233	0.001	0.001
0.0025	0.1258	0.0007	0.0005
0.0062	0.0127	0.0185	0.0051
0.0097	0.0124	0.0194	0.0049
0.0128	0.0114	0.0166	0.0045
0.0051	0.0093	0.0162	0.0044
0.0082	0.018	0.0188	0.0059
0.0058	0.0127	0.0148	0.0046
0.001	0.1733	0.001	0.0003
0.0022	0.146	0.0004	0.0002
0.0015	0.168	0.0006	0.0005
0.0014	0.1803	0.0007	0.0006
0.0049	0.1799	0.0004	0.0003
0.0018	0.1746	0.0003	0.0003
0.0014	0.2098	0.0006	0.0003
0.0007	0.2419	0.0006	0.0004
0.0007	0.2656	0.0006	0.0003
0.0012	0.1408	0.0001	0.0002
0.0013	0.2188	0.0006	0.0002
0.0011	0.2092	0.0002	0.0002

Phosphocreatine	Pyruvate	Succinate	Threonine
0.0244	0.0744	0.1448	0.0146
0.0239	0.0731	0.1512	0.0254
0.0265	0.0843	0.1851	0.014
0.0192	0.0817	0.1636	0.0665
0.026	0.0761	0.1518	0.0145
0.0243	0.0795	0.1603	0.0241
0.025	0.0039	0.0449	0.0221
0.0238	0.004	0.0428	0.0262
0.0237	0.0033	0.0434	0.0147
0.0268	0.0044	0.0479	0.0183
0.0246	0.0047	0.0418	0.0274
0.022	0.0043	0.0421	0.0213
0.0261	0.0168	0.0087	0.0117
0.0242	0.016	0.009	0.0107
0.0234	0.017	0.0075	0.0096
0.0219	0.0151	0.0083	0.007
0.0218	0.0161	0.0066	0.007
0.0255	0.0177	0.0073	0.019
0.0268	0.3164	0.2881	0.0208
0.0402	0.5064	0.4476	0.0251
0.0283	0.3597	0.3191	0.0133
0.0267	0.356	0.2972	0.034
0.0339	0.4927	0.4341	0.0215
0.0266	0.3609	0.3124	0.0197
0.0256	0.0244	0.0147	0.0241
0.0236	0.0208	0.0129	0.0152
0.0259	0.0237	0.0138	0.0189
0.0255	0.0246	0.0147	0.0189
0.0248	0.0258	0.0158	0.0177
0.0243	0.0241	0.0149	0.0157
0.0255	0.004	0.0126	0.006
0.0277	0.0029	0.0146	0.0008
0.0294	0.0036	0.0161	0.0075
0.017	0.0017	0.0084	0.0057
0.0256	0.003	0.0135	0.0103
0.0204	0.0019	0.0108	0.0018

Tyrosine	Valine
0.0001	0.0005
0.0001	0.0004
0.0001	0.0003
0.0001	0.0002
0.0001	0.0002
0.0002	0.0007
0.0026	0.0213
0.0029	0.0228
0.003	0.0204
0.0027	0.0218
0.0024	0.0224
0.0023	0.0198
0.0002	0.0019
0.0006	0.0021
0.0005	0.0018
0.0011	0.0017
0.0011	0.0019
0.0006	0.0022
0.0054	0.0167
0.0064	0.0233
0.0048	0.0163
0.005	0.0154
0.007	0.0211
0.0053	0.0145
0.0001	0.0009
0.0001	0.001
0.0001	0.0011
0.0003	0.0008
0.0001	0.0008
0.0001	0.0009
0.0002	0.0009
0.0004	0.0009
0.0004	0.001
0.0001	0.0006
0.0002	0.0009
0.0002	0.0006
